# Polycystic ovarian disease and associated factors

**DOI:** 10.1186/s41043-016-0047-z

**Published:** 2016-04-14

**Authors:** Jyoti Thulkar, Shalini Singh

**Affiliations:** Scientist, Division of Publication & Information Indian Council of Medical Research, New Delhi, India

Sir,

The article by Ebrahimi-Mamaghani M et al. is quite informative and focuses on insulin resistance and lipid profile [1]. We work in a research organization which supports extramural and intramural research and would therefore like to know more on some aspects of this study as stated below.

1. The authors state that lipid abnormality may occur in polycystic ovary syndrome (PCOS) irrespective of insulin resistance (IR) in overweight and obese women. Indian literature showed that about one fourth of PCOS women have normal body mass index (BMI) [2]. PCOS women with normal BMI are very difficult to treat and monitor. Does the lipid abnormality exit in this category of women also, and will it help in monitoring of the PCOS women with normal BMI?
2. The main focus of this study is on overweight and obese PCOS women. This classification is dependent on BMI, but waist circumference has not been considered. However, many studies indicate that waist circumference is a more sensitive parameter to define obesity [3, 4]. We would like to know whether authors have found any relationship between waist circumference, hyperlipidemia, and IR. This information is important in monitoring response of the patients to treatment. Reduction in waist circumference is a non-invasive indicator of successful treatment and will be useful in low-resource settings also.
3. In the present study, prevalence of IR is 36.7 %, which is higher than that of previously reported studies [5]. Obesity is the underlying cause of the development of IR. Is this high prevalence reported largely because of the overweight and obese women? We would like to know whether the authors recommend IR documentation in all the PCOS women irrespective of BMI and whether IR investigation would be helpful in monitoring treatment in PCOS women irrespective of BMI.
4. Age has been reported as an important factor in the development of PCOS. IR and obesity also develop over a period of time and increase with age [6]. We would request the authors to discuss the relationship of IR in different age groups in a study population.
5. The authors have reported lower testosterone level among patients with IR. What would be the suggested underlying mechanism for this finding? Did they find any relationship with estrogen level? Free testosterone level has a positive correlation with HOMA-IR in postmenopausal women but not in premenopausal women [7]. Postmenopausal women are considered to have lower estrogenic levels than premenopausal women. In this respect, knowing estrogen level in PCOS women is important.
